# Mixed *Candida albicans* strain populations in colonized and infected mucosal tissues

**DOI:** 10.1111/j.1567-1364.2008.00438.x

**Published:** 2008-09-15

**Authors:** Mette D Jacobsen, Amanda D Duncan, Judith Bain, Elizabeth M Johnson, Julian R Naglik, Duncan J Shaw, Neil AR Gow, Frank C Odds

**Affiliations:** 1 Aberdeen Fungal Group, Institute of Medical SciencesAberdeen, UK; 2 Mycology Reference Laboratory, KingsdownBristol, UK; 3 Department of Oral Immunology, King's College London Dental InstituteLondon UK

**Keywords:** *Candida albicans*, MLST, strain typing, epidemiology

## Abstract

Multilocus sequence typing of six *Candida albicans* colonies from primary isolation plates revealed instances of colony-to-colony microvariation and carriage of two strain types in single oropharyngeal and vaginal samples. Higher rates of colony variation in commensal samples suggest selection of types from mixed populations either in the shift to pathogenicity or the response to antifungal treatment.

## Introduction

*Candida albicans* is the most common fungal species found as a gut commensal and opportunistic human pathogen. By multilocus sequence typing (MLST) of two or more sets of *C. albicans* isolates from 44 patients, we showed previously that microvariation, typically seen in the form of apparent gain and loss of sequence heterozygosity in one or more of the seven genes used for MLST, was demonstrable in 36 of the patients, and strain replacement was evidenced unequivocally in one patient between two hospital admissions (Odds *et al.*, 2006). The observation of microvariation between multiple *C. albicans* isolates from single sources has been demonstrated previously by other genotyping methods (Schroppel *et al.*, 1994; Lockhart *et al.*, 1995; Pujol *et al.*, 1999; Chong *et al.*, 2003; Samaranayake *et al.*, 2003a, b; Forche *et al.*, 2005; Sampaio *et al.*, 2005). We suggested that one explanation for the high level of microvariation encountered was that natural *C. albicans* populations *in vivo* comprise a mixture of closely related strain types, with a high level of genetic diversity maintained by mechanisms such as recombination and chromosomal ploidy shifts (Odds *et al.*, 2006).

Phenotypic evidence for mixed *C. albicans* strain populations *in vivo* exists in terms of variations of antifungal susceptibility (Johnson *et al.*, 1995; Le Guennec *et al.*, 1995) and of colony form (Hellstein *et al.*, 1993) between individual colonies in the primary isolation cultures. Hellstein *et al.* (1993) used Ca3 DNA fingerprinting to examine pairs of different colony forms among oral isolates from four patients and showed that one of the four patients carried two different, but related types. Le Guennec *et al.* (1995) used multilocus enzyme electrophoresis (MLEE) to characterize 10-colony sets cloned from 10 primary oral isolation plates for four AIDS patients. They found no MLEE variation among 40 colonies on four isolation plates from one patient, while MLEE differences consistent with microvariation were found among colonies from the other six plates.

To explore the prevalence of mixed *C. albicans* populations among individual patients further, we undertook a prospective survey by MLST of six individual *C. albicans* colonies chosen at random from 32 primary isolation plates. We obtained primary oral isolation plates, presumed to represent commensal carriage, from healthy volunteers and primary oral and vaginal isolation plates from patients with symptomatic oral and vaginal *C. albicans* infections. MLST for six randomly selected colonies on each isolation plate revealed that microvariation differences between colonies occurred more commonly in the commensal samples than the samples from infected patients. One of the 12 healthy volunteers was found to carry two entirely different strain types on the same isolation plate.

## Materials and methods

Mouthwash samples positive for *C. albicans* were obtained from 12 undergraduate student volunteers who provided samples anonymously. The students rinsed their mouths with 10 mL of sterile distilled water, returned the fluid to a sterile container, and 100 µL was plated on Sabouraud agar. Plates bearing from 6 to 50 yeast colonies (mode=10 colonies) were selected for further study. From the yeast growth, six well-separated colonies were chosen and propagated separately on Sabouraud agar slants. Samples from 10 female patients with symptomatic vaginitis and from 10 patients (five females) with various forms of oral *Candida* infection were handled similarly, except that the yeast growth came from plates inoculated from vaginal swabs and whole saliva samples, respectively. For the patients with vaginitis, the separate colonies had been streaked out from confluent yeast growth. For those with oral infection, the salivary *C. albicans* counts ranged from 20 to >10^4^ yeasts mL^-1^ saliva, with most samples containing >10^3^ yeasts mL^-1^: these samples were plated with dilutions of saliva to provide the separated colonies sampled for this study. The antifungal treatment status of the patients at the time of sampling was unknown in most cases.

Presumptive identification of the isolates as *C. albicans* was based on colony colour on CHROMagar *Candida* (Odds & Bernaerts, 1994) and confirmed using PCR with primers that amplified the *ITS1* region of ribosome-encoding DNA, which also designated the ATP-binding cassette (ABC) type of each isolate (McCullough *et al.*, 1999). All 192 colonies were further typed by MLST and for homozygosity at the mating-type locus as described previously (Bougnoux *et al.*, 2003; Tavanti *et al.*, 2003). The MLST data, representing 1344 bidirectional sequence determinations, were assigned to genotypes for the seven loci sequenced and to diploid sequence types (DSTs) by reference to the Internet database for *C. albicans* MLST (http://test1.mlst.net/).

## Results and discussion

Details of the 32 subjects whose primary isolation plates were the sources of six random colonies for strain typing are given in Table 1. For five of the 12 healthy volunteers, all six colonies from mouthwash isolation plates were the same DST and ABC type and all were heterozygous at the MTL. For Student04, five colonies were indistinguishable by DST and ABC type, but the sixth colony was a different DST and ABC type and even represented a different clade of strains from the other five colonies (Table 1). This result was interpreted as evidence of carriage of two unrelated strain types in a single individual. For Student10, one colony was a different but closely related DST from the other five colonies, and its ABC type (A) differed from that of the other five colonies (type C). This equivocal result may indicate either a microvariation in strain types or carriage of distinct types in a single individual. For the remaining five primary isolations from the healthy volunteers, either one or two colonies differed from the remainder in the sequence for just one of the seven DNA fragments used for MLST, a level of disparity we regard as indicating sequence microvariation. ABC types and MTL data were the same for all six colonies tested despite the variations in DST.

**Table 1:** Details of sources of *Candida albicans* isolates and results of MLST, ABC and MTL typing

		Strain typing data				
				
Subject reference	Details	No. of colonies	DST	Clade*	ABC type	MTL type
Student01	Healthy volunteer	6	1029	8	A	*a/*α
Student02	Healthy volunteer	5	845	2	A	*a/*α
		1	844	2	A	*a/*α
Student03	Healthy volunteer	6	846	1	A	*a/*α
Student04	Healthy volunteer	5	766	1	B	*a/*α
		1	497	2	A	*a/*α
Student05	Healthy volunteer	6	857	2	A	*a/*α
Student06	Healthy volunteer	2	4	2	A	*a/*α
		4	232	2	A	*a/*α
Student07	Healthy volunteer	4	1025	1	B	*a/*α
		1	1026	1	B	*a/*α
		1	1082	1	B	*a/*α
Student08	Healthy volunteer	5	1024	2	A	*a/*α
		1	1083	2	A	*a/*α
Student09	Healthy volunteer	4	1027	1	A	*a/*α
		1	1028	1	A	*a/*α
		1	1009	1	A	*a/*α
Student10	Healthy volunteer	5	367	1	C	*a/*α
		1	766	1	A	*a/*α
Student11	Healthy volunteer	6	155	2	A	*a/*α
Student12	Healthy volunteer	6	855	6	B	*a/*α
Vag01	Vaginitis patient	6	1014	1	A	*a/*α
Vag02	Vaginitis patient	6	155	2	A	*a/*α
Vag03	Vaginitis patient	5	322	1	A	*a/a*
		1	1013	1	A	*a/a*
Vag04	Vaginitis patient	6	365	8	A	*a/*α
Vag05	Vaginitis patient	3	1144	1	A	*a/*α
		1	1143	1	A	*a/*α
		1	1145	1	A	*a/*α
		1	1146	1	A	*a/*α
Vag06	Vaginitis patient	5	572	1	B	*a/*α
		1	584	1	B	*a/*α
Vag07	Vaginitis patient	6	344	3	A	*a/*α
Vag08	Vaginitis patient	6	60	1	A	*a/*α
Vag09	Vaginitis patient	6	277	1	A	*a/*α
Vag10	Vaginitis patient	6	1151	1	A	*a/*α
Oral01	Lichen planus, depapillated tongue	6	277	1	A	*a/*α
Oral02	Sjogren's syndrome	6	1147	1	A	*a/*α
Oral03	Mucous membrane pemphigoid and *C. albicans* infection	5	1148	2	A	*a/*α
		1	1149	2	A	*a/*α
Oral04	Lichen planus	6	1150	4	B	*a/*α
Oral05	Oral infection; fluconazole Rx	6	1051	1	A	*a/*α
Oral06	*Candida* elements in biopsy	6	1076	1	A	*a/*α
Oral07	Recurrent aphthous stomatitis	6	1077	–†	A	*a/*α
Oral08	Sjogren's syndrome	5	1078	9	A	*a/*α
		1	1079	9	A	*a/*α
Oral09	Oral *Candida* with lichen planus	5	1080	2	A	*a/*α
		1	1081	2	A	*a/*α
Oral10	Recurrent oral *Candida* infection	6	1052	2	A	*a/*α

* Determined according to reference Odds *et al.* (2007).

† DST 1077 clustered close to IHEM20439, which was a singleton, not assignable to a clade in reference Odds *et al.* (2007).

Among the 10 primary isolation plates from patients with vaginitis, two examples of one colony DST differing from the other five tested were found (Vag03 and Vag06, Table 1). For patient Vag05, four different but closely related DSTs were found among the six colonies tested. This represents the most disparate example of microvariation encountered among all 32 sets of isolates: all ABC and MTL data for this patient were the same. Among the 10 isolation plates from patients with oral *Candida* infections, three instances of single-colony DST differences were found (Oral03, Oral08 and Oral09, Table 1). ABC and MTL data were indistinguishable, patient per patient, for the colonies isolated from oral and vaginal infections.

From the 32 subjects, overall, we isolated strains representing seven *C. albicans* clades (Odds *et al.*, 2007) plus one singleton. Discounting Student04, who carried strains from clades 1 and 2, 15 patients had isolates from clade 1, nine from clade 2, two from clade 8 and one each from clades 3, 4, 6 and 9 and a singleton (Table 1). Clade 1 is the most ubiquitous *C. albicans* clade, containing one-third of all isolates among 1391 studied from all global sources (Odds *et al.*, 2007), and 70% of the 171 clade 2 isolates in the same study came from the UK. Because all the isolates in the present study were of UK origin, the heavy representation of isolates from clades 1 and 2 is consistent with existing epidemiologic information and the clade distribution of isolates in our sample appears to be typical for a set from 32 subjects in the UK.

The finding of a difference in ABC type between colonies for one of the isolates (from Student10) was unusual but not entirely unexpected; previously we found variability in ABC types within two sets of isolates from 43 sources (Odds *et al.*, 2006).

Examples of DNA sequence microvariation, also called ‘micro-evolution’, between colonies have been evidenced previously by DNA fingerprinting (Hellstein *et al.*, 1993) and by MLEE (Le Guennec *et al.*, 1995), and microvariation after measured numbers of population generations has been documented as a consequence of exposure of *C. albicans* to azole antifungal agents (Cowen *et al.*, 2000, 2001). Our data represent a much larger sample size than was used previously to examine intercolony DNA variations and exemplify further the high level of genetic plasticity of *C. albicans* noted in many studies, which may serve as a substitute for generation of diversity in the absence of a meiotic sexual cycle. MLST results usually remain stable for a single colony isolate of *C. albicans* propagated infrequently, but our unpublished results show that MLST types can alter with prolonged exposure to antifungal agents, as already found by Cowen *et al.* (2000, 2001). We also have preliminary (unpublished data) evidence of selective overgrowth of some DSTs when different strains are cocultured in competition.

Our data showed a higher prevalence of colony-to-colony variation in primary isolations from healthy volunteers (six of 12 subjects showing microvariation based on DST, plus a seventh subject with two definitively different strain types in the single sample) than in the samples from patients with superficial infections (six of 20 subjects showed microvariation based on DST). If a colonizing population of *C. albicans* comprises a mixture of types, which was the case for 7/12 of our healthy volunteers, it seems reasonable to hypothesize that increased cell numbers and epithelial invasion associated with superficial infections may result from the selective proliferation of a single subtype, or a set of fewer subtypes, that was present in the mixed commensal population before invasive infection. This conclusion is qualified by the fact that the proportion of all isolated colonies tested was higher for the commensal samples, where numbers of colonies initially isolated were small compared with the high saliva counts and confluent growth from swabs for the patients. Because the antifungal treatment status was unknown for the majority of the patients, we cannot distinguish between selection of a less variable colony population arising from differences in invasiveness and selection in response to antifungal exposure.

## References

[b1] Bougnoux ME, Tavanti A, Bouchier C (2003). Collaborative consensus for optimized multilocus sequence typing of *Candida albicans*. J Clin Microbiol.

[b2] Chong PP, Lee YL, Tan BC, Ng KP (2003). Genetic relatedness of *Candida* strains isolated from women with vaginal candidiasis in Malaysia. J Med Microbiol.

[b3] Cowen LE, Sanglard D, Calabrese D, Sirjusingh C, Anderson JB, Kohn LM (2000). Evolution of drug resistance in experimental populations of *Candida albicans*. J Bacteriol.

[b4] Cowen LE, Kohn LM, Anderson JB (2001). Divergence in fitness and evolution of drug resistance in experimental populations of *Candida albicans*. J Bacteriol.

[b5] Forche A, May G, Magee PT (2005). Demonstration of loss of heterozygosity by single-nucleotide polymorphism microarray analysis and alterations in strain morphology in *Candida albicans* strains during infection. Eukaryot Cell.

[b6] Hellstein J, Vawterhugart H, Fotos P, Schmid J, Soll D (1993). Genetic similarity and phenotypic diversity of commensal and pathogenic strains of *Candida albicans* isolated from the oral cavity. J Clin Microbiol.

[b7] Johnson EM, Warnock DW, Luker J, Porter SR, Scully C (1995). Emergence of azole drug resistance in *Candida* species from HIV-infected patients receiving prolonged fluconazole therapy for oral candidosis. J Antimicrob Chemother.

[b8] Le Guennec R, Reynes J, Mallie M, Pujol C, Janbon F, Bastide JM (1995). Fluconazole-and itraconazole-resistant *Candida albicans* strains from AIDS patients multilocus enzyme electrophoresis analysis and antifungal susceptibilities. J Clin Microbiol.

[b9] Lockhart SR, Fritch JJ, Meier AS, Schroppel K, Srikantha T, Galask R, Soll DR (1995). Colonizing populations of *Candida albicans* are clonal in origin but undergo microevolution through C1 fragment reorganization as demonstrated by DNA fingerprinting and C1 sequencing. J Clin Microbiol.

[b10] McCullough MJ, Clemons KV, Stevens DA (1999). Molecular and phenotypic characterization of genotypic *Candida albicans* subgroups and comparison with *Candida dubliniensis* and *Candida stellatoidea*. J Clin Microbiol.

[b11] Odds FC, Bernaerts R (1994). CHROMagar Candida, a new differential isolation medium for presumptive identification of clinically important *Candida* species. J Clin Microbiol.

[b12] Odds FC, Davidson AD, Jacobsen MD (2006). *Candida albicans* strain maintenance, replacement, and microvariation demonstrated by multilocus sequence typing. J Clin Microbiol.

[b13] Odds FC, Bougnoux M-E, Shaw DJ (2007). Molecular phylogenetics of *Candida albicans*. Eukaryot Cell.

[b14] Pujol C, Joly S, Nolan B, Srikantha T, Soll DR (1999). Microevolutionary changes in *Candida albicans* identified by the complex Ca3 fingerprinting probe involve insertions and deletions of the full-length repetitive sequence RPS at specific genomic sites. Microbiol – UK.

[b15] Samaranayake YH, Samaranayake LP, Dassanayake RS, Yau JYY, Tsang WK, Cheung BPK, Yeung KWS (2003a). ‘Genotypic shuffling’ of sequential clones of *Candida albicans* in HIV-infected individuals with and without symptomatic oral candidiasis. J Med Microbiol.

[b16] Samaranayake YH, Samaranayake LP, Yau JYY, Dassanayake RS, Li TKL, Anil S (2003b). Phenotypic diversity of oral *C. albicans* isolated on single and sequential visits in an HIV-infected Chinese cohort. APMIS.

[b17] Sampaio P, Gusmao L, Correia A (2005). New microsatellite multiplex PCR for *Candida albicans* strain typing reveals microevolutionary changes. J Clin Microbiol.

[b18] Schroppel K, Rotman M, Galask R, MAC K, Soll DR (1994). Evolution and replacement of *Candida albicans* strains during recurrent vaginitis demonstrated by DNA fingerprinting. J Clin Microbiol.

[b19] Tavanti A, Gow NAR, Senesi S, Maiden MCJ, Odds FC (2003). Optimization and validation of multilocus sequence typing for *Candida albicans*. J Clin Microbiol.

